# Supplementary material for: DandD: Efficient measurement of sequence growth and similarity
Source: iScience. 2024 Feb 1;27(3):109054. doi: 10.1016/j.isci.2024.109054 (PMC10867639; doi:10.1016/j.isci.2024.109054)
Supplement: Document S1. Figure S1 [file mmc1.pdf]

iScience, Volume *27*

Supplemental information

DandD: Efficient measurement of sequence growth and similarity

Jessica K. Bonnie, Omar Y. Ahmed, and Ben Langmead

Supplementary Information

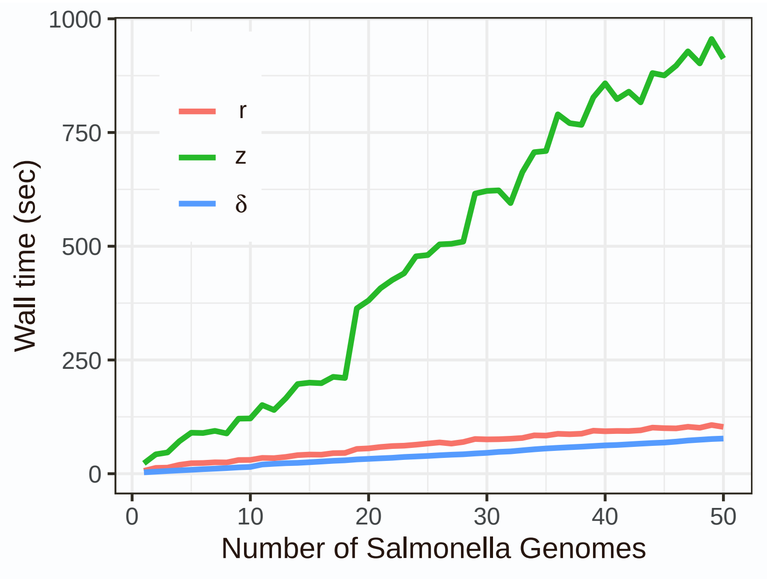

Figure S1: **Wall clock time required to compute δ, r, and z on the arbitrary ordering of 50 Salmonella genomes examined in Figure 2** All experiments used a single thread of execution. We note that these benchmarks use a particular set of tools to compute each measure. It does not necessarily convey the inherent difficulty of computing the measures. A related caveat is that z is computed using a series of tools that first computes a prefix-free parse, from which r can be immediately inferred. In this way, the method we used to estimate z was bound to use more wall time than the method used to estimate r. A method that computes z directly, not by way of the prefix-free parse, might be more performant.
